# Associations between a normal-range free thyroxine concentration and ovarian reserve in infertile women undergoing treatment via assisted reproductive technology

**DOI:** 10.1186/s12958-024-01226-6

**Published:** 2024-06-22

**Authors:** Qiaoling Zhang, Dandan Zhang, Haoyuan Liu, Jinyun Fu, Li Tang, Meng Rao

**Affiliations:** 1 Department of Reproduction and Genetics, The First Affiliated Hospital of Kunming Medical University, No. 295 Xichang Road, Kunming, 650032 China; 2 Department of Endocrinology, The First Affiliated Hospital of Kunming Medical University, No. 295 Xichang Road, Kunming, 650032 China

**Keywords:** Ovarian reserve, Normal thyroid function, Free thyroxine, anti-Müllerian hormone

## Abstract

**Background:**

Some recent studies have shown that female subclinical hypothyroidism (SCH) is associated with diminished ovarian reserve (DOR). In this study, we aimed to investigate whether serum-free thyroxine (fT4) concentrations within the reference range are associated with ovarian reserve in women.

**Methods:**

This cross-sectional study included 4933 infertile women with normal-range fT4 concentrations who received assisted reproductive technology treatment in our clinic. The data of women in different fT4 concentration tertiles (namely 12–15.33, 15.34–18.67, and 18.68–22 pmol/L) were compared with ovarian reserve markers, namely the anti-Müllerian hormone (AMH) concentration, the antral follicle count (AFC), and the number of aspirated oocytes. The primary outcomes were the AMH concentration and the risk of DOR, diagnosed as an AMH concentration < 1.1 ng/mL.

**Results:**

The average ages of women in the low-normal, middle-normal, and high-normal fT4 tertiles were 33.20 (standard deviation [SD]: 5.11), 32.33 (SD: 5.13), and 31.61 (SD: 5.10) years, respectively (*p* < 0.0001). AMH concentrations (adjusted mean: 3.32 [95% confidence interval {CI}: 3.16 to 3.50] vs. 3.51 [3.40 to 3.62] vs. 3.64 [3.50 to 3.80] ng/mL, *p* = 0.022) were significantly different between the fT4 concentration tertiles. The risk of DOR was significantly increased in the low-normal (adjusted odds ratio: 1.61 [95% CI: 1.01 to 2.58]) and middle-normal (1.47 [95% CI: 1.00 to 2.16]) tertiles compared with the high-normal tertile. Subgroup analysis showed that AMH concentrations were significantly different among the fT4 concentration tertiles in women aged < 35 years (adjusted mean: 3.94 [95% CI: 3.70 to 4.20] vs. 4.25 [4.11 to 4.39] vs. 4.38 [4.18 to 4.58], *p* = 0.028), whereas this difference was not significant in women aged ≥ 35 years (*p* = 0.534). The general additive models using fT4 as a continuous variable indicated that a lower fT4 concentration within the normal range was significantly associated with a lower AMH concentration (*p* = 0.027), a lower AFC (*p* = 0.018), a lower number of aspirated oocytes (*p* = 0.001), and a higher risk of DOR (*p* = 0.007).

**Conclusion:**

Low-normal fT4 concentrations are associated with lower ovarian reserve in infertile women.

## Background

Thyroid function is critical for the regulation of reproductive function in women of childbearing age. Thyroid dysfunction in women is associated with menstrual disorder, diminished ovarian reserve (DOR), and an increased risk of miscarriage and preterm birth [1, 2]. Thyroid hormones and their receptors exist in multiple components of ovarian tissues, including follicular fluid [1], theca cells [3], and granulosa cells [4], which indicates their important roles in the regulation of ovarian function. Animal studies have suggested that thyroid hormones are critical for follicle-stimulating hormone-induced preantral follicular development [5] and that inadequate secretion of thyroid hormones can disturb folliculogenesis [6].

Our previous studies have demonstrated that subclinical hypothyroidism (SCH), defined as an increased serum thyroid-stimulating hormone (TSH) concentration that coexists with a normal serum-free thyroxine (fT4) concentration, is associated with DOR in women aged 35 years or older [7] and is significantly associated with an increased risk of an abnormal DNA fragmentation index in infertile men [8]. An elevated TSH concentration has been reported to be associated with a decreased anti-Müllerian hormone (AMH) concentration, disturbed folliculogenesis [9], and increased risks of miscarriage and preterm birth [10]. Notably, patients with SCH in our previous studies had lower fT4 concentrations than those with euthyroidism (16.31 [interquartile range {IQR}: 2.81] vs. 16.94 [IQR: 3.00] pmol/L) [7]. Indeed, elevated or decreased thyroid function parameters, even within their normal ranges, have been reported to impact serum cholesterol levels, blood pressure [11], and atrial fibrillation [12]. Our recent study also showed that low-normal paternal fT4 concentrations were associated with worse pregnancy outcomes of assisted reproductive technology (ART) [13]. However, it remains unclear whether variations in women’s thyroid function within the normal range are associated with ovarian reserve.

In the present study, we aimed to investigate the associations between normal-range fT4 concentrations and ovarian reserve in a large population of infertile women who were receiving ART treatment.

## Methods

### Study design and population

This cross-sectional study was carried out at the Reproductive Medical Center of the First Affiliated Hospital of Kunming Medical University from March 2016 to April 2023. Patients who received ART treatments during the study period were potentially eligible. The antral follicle count (AFC) was determined by transvaginal ultrasonography examinations on days 2–4 of an unstimulated menstrual cycle. Blood samples also collected on days 2–4 of the menstrual cycle for analysis on the same day were used to measure the serum levels of AMH concentration and markers of thyroid function. The demographic and clinical data of the patients who underwent intrauterine insemination, in vitro fertilization and embryo transfer (IVF-ET), or intracytoplasmic sperm injection (ICSI) were recorded in a database.

**Fig. 1:**
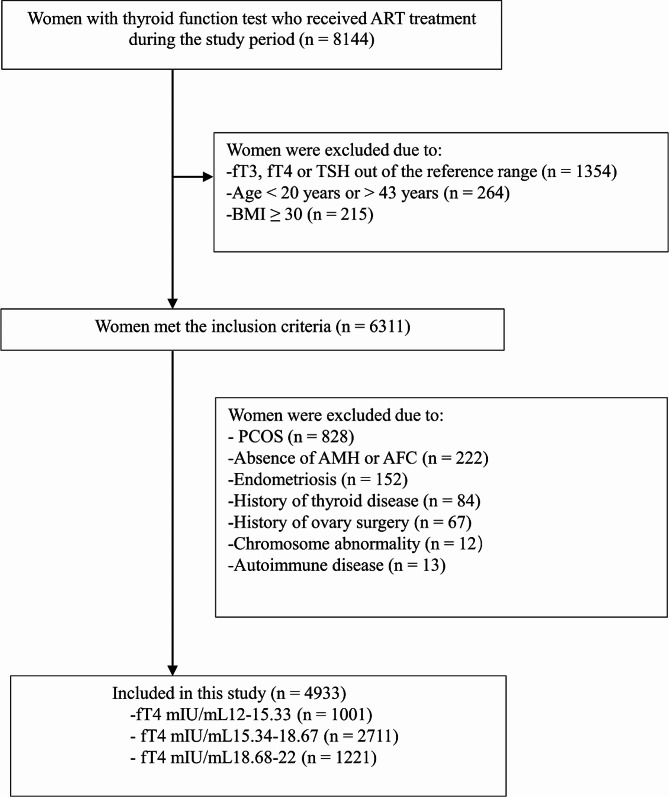
Flowchart of the participant selection in this study TAI: thyroid autoimmunity; ART: assisted reproductive technology; PCOS: polycystic ovarian syndrome; AMH: anti-Müllerian hormone; AFC: antral follicle count

The inclusion criteria for participants were as follows: (1) female patients aged 20–43 years; (2) a body mass index (BMI) less than 30 kg/m^2^; (3) treatment with IVF/ICSI; and (4) normal concentrations of free triiodothyronine (fT3), fT4, and serum thyrotropin (TSH). The exclusion criteria were women who were diagnosed with (1) polycystic ovarian syndrome, (2) endometriosis, (3) overt thyroid dysfunction or those who had (4) a history of thyroid disease (treated or not), (5) a history of ovarian surgery or ovarian tumor, (6) chromosomal abnormality. Finally, the data of 4933 women were included in the data analysis (Fig. 1).

The patients were divided into three groups based on normal fT4 concentration tertiles in women: the low-normal tertile (12.00–15.33 pmol/L), middle-normal tertile (15.34–18.67 pmol/L), and high-normal tertile (18.68–22.00 pmol/L) [13]. The differences in AMH concentration and the risk of DOR, defined as an AMH concentration < 1.1 ng/mL [14–16], between the fT4 concentration tertile groups were analyzed. The AFC was compared between the three fT4 concentration groups.Last, the number of aspirated oocytes, which reflects the ovarian reserve and the response to ovarian stimulation, was also compared between the three fT4 concentration groups.

The primary outcomes of this study were the AMH concentration and the risk of DOR. The secondary outcomes were the AFC and the number of aspirated oocytes.

### Measurement of the AMH concentration

Serum AMH concentrations were measured using a commercially available enzyme-linked immunosorbent assay (ELISA) kit (Kangrun Biotech, Guangzhou, China) and a plate reader (EXL808; BioTek Instruments, Shanghai, China). The linear range of the kit is 0.07–18 ng/ml, *r* ≥ 0.9900, the minimum detection limit ≤ 0.06 ng/mL. and the intra- and inter-assay coefficients of variation (CVs) were ≤ 15% and ≤ 8%, respectively.

### Measurement of thyroid function

We measured serum fT3, fT4, TSH, thyroperoxidase antibody (TPO-Ab), and thyroglobulin antibody (Tg-Ab) concentrations using ELISAs and a Cobas E601 analyzer (Roche), as described previously [17]. We used the manufacturer-recommended reference ranges as follows: 0.27–4.20 µIU/mL for TSH, 3.1–6.8 pmol/L for fT3, 12–22 pmol/L for fT4, < 115.0 IU/mL for Tg-Ab, and < 34 IU/mL for TPO-Ab. The ranges of intra-assay CVs were 1.1–3.0% for TSH, 1.3–6.5% for fT3, 1.6–5.0% for fT4, 1.8–2.1% for Tg-Ab, and 2.8–4.8% for TPO-Ab. The ranges of inter-assay CVs were 3.2–7.2% for TSH, 1.6–7.2% for fT3, 1.9–6.3% for fT4, 4.6–5.1% for Tg-Ab, and 3.5–6.1% for TPO-Ab. TPO-Ab concentrations ≥ 34 IU/mL and Tg-Ab concentrations ≥ 115 IU/mL were considered positive. Thyroid autoimmunity (TAI) was diagnosed when the patient tested positive for TPO-Ab or Tg-Ab.

### Measurement of the AFC

We evaluated the ovarian AFCs on days 2–4 of an unstimulated menstrual cycle. First, transvaginal ultrasound was performed jointly by two well-trained reproductive endocrinologists to estimate the number of follicles. The AFC for each patient was then calculated as the sum of antral follicles (2–9 mm in diameter) in both ovaries in the early follicular phase.

### Ovarian stimulation and oocyte aspiration

The number of aspirated oocytes is one of the indexes uesd to evaluate ovarian reserve function, and the number of aspirated oocytes obtained varies with different ovarian stimulation protocols. Therefore, it is necessary to adjust the effects of different ovarian stimulation protocols when assessing oavrian reserve function. The patients were treated with different ovarian stimulation protocols, including long-term gonadotropin-releasing hormone (GnRH) agonist (long protocol, *n* = 2189), GnRH antagonist (antagonist protocol, *n* = 1828), the mild stimulation protocol (*n* = 491), and other protocols (*n* = 366) according to their conditions. The details of the therapeutic regimens of different stimulation protocols are described in previous studies [18, 19]. Recombinant human chorionic gonadotropin (HCG; 250 mg, Ovidrel; Serono) was administered when two leading follicles reached an average diameter of 18 mm. Oocytes were then retrieved transvaginally 34–36 h after HCG administration by using a single lumen needle attached to a syringe to aspirate the follicles under transvaginal ultrasound guidance. Finally, the oocytes were placed in a culture dish and confirmed under a stereomicroscope.

### Statistical analysis

The demographic and clinical data of the patients in the three fT4 concentration groups were compared using a one-way analysis of variance for normally distributed continuous variables, the Kruskal–Wallis H test for non-normally distributed continuous variables, and Pearson’s chi-square test for categorical variables. Multivariable generalized linear models with linear (AMH concentration as the dependent variable), logit (the risk of DOR as the dependent variable), and Poisson (the AFC and the number of aspirated oocytes as the dependent variables) links were used to analyze the associations between fT4 concentrations and ovarian reserve markers. The models were adjusted for women’s age, BMI, TSH concentration, infertility type, infertility diagnosis, infertility duration, and TAI positivity when analyzing the AMH concentration, risk of DOR, and AFC. To reduce skewness, AMH concentrations were subjected to log_10_ transformation before performing regression analyses, as described previously [20, 21]. The models were further adjusted for the ovulation stimulation protocol, gonadotropin dose, and gonadotropin duration when analyzing the number of aspirated oocytes.

To investigate the potential effect of the patient’s age on the associations between the fT4 concentration tertiles and ovarian reserve, we performed a regression analysis stratified by age (< 35 or ≥ 35 years) using a generalized linear model. The associations of fT4 concentrations with the AMH concentration, risk of DOR, AFC, and number of aspirated oocytes were also analyzed using a general additive model (GAM) and smooth curve fitting. Confounding factors were adjusted in all of the abovementioned analyses. All of the statistical tests were two-sided, and a *p* value < 0.05 was considered statistically significant. SPSS 26.0 (SPSS, IBM Inc., Chicago, IL USA), EmpowerStats 4.1 (X&Y Solutions Inc., Boston, MA, USA), and R 4.3.1 (packages mgcv and ggplot2, the R Foundation) were used for data analysis.

## Results

### Characteristics of the enrolled participants

A total of 8144 patients received ART treatment during the study period. Following application of the inclusion and exclusion criteria, 4933 patients were included in the final analysis. Of these, 1001, 2711, and 1221 women had fT4 concentrations in the low-normal, middle-normal, and high-normal tertiles, respectively. In these three groups, the mean ages of women were 33.20 (SD: 5.11), 32.33 (SD: 5.13), and 31.61 (SD: 5.10) years, respectively (*p* < 0.0001), and the mean BMIs were 22.80 (SD: 2.80), 22.11 (SD: 2.85), and 21.76 (SD: 2.95) kg/m^2^, respectively (*p* < 0.0001). The incidence rates of DOR in the three groups were 22.28%, 18.92%, and 15.40%, respectively (*p* = 0.0002). The median AMH concentrations, AFCs, and numbers of aspirated oocytes were significantly different between the three groups (2.61 [IQR: 1.25 to 4.41] vs. 2.86 [IQR: 1.51 to 4.91] vs. 3.16 [IQR: 1.73 to 5.39] ng/mL; 12 [IQR: 7 to 16] vs. 13 [IQR: 8 to 17] vs. 13 [IQR: 9 to 18]; and 9 [IQR: 4 to 14] vs. 10 [IQR: 5 to 15] vs. 11 [IQR:6 to 16], respectively; all *p* < 0.0001). The demographic and clinical data of the included patients are presented in Table 1.

**Table 1:** Characteristics of included participants

Characteristics	Maternal fT4 tertiles (pmol/L)			*P*-Value
	12.00-15.33 (*N* = 1001)	15.34–18.67 (*N* = 2711)	18.68-22.00 (*N* = 1221)	
Age, years, mean (SD)	33.20 (5.11)	32.33 (5.13)	31.61 (5.10)	<0.0001
Age group, n (%)				<0.0001
<35years	598 (59.74)	1797 (66.29)	887 (72.65)	
≥ 35years	403 (40.26)	914 (33.71)	334 (27.35)	
BMI, kg/m2, mean (SD)	22.80 (2.80)	22.11 (2.85)	21.76 (2.95)	<0.0001
AMH, ng/ml, median (IQR)	2.61 (1.25 to 4.41)	2.86 (1.51 to 4.91)	3.16 (1.73 to 5.39)	<0.0001
AFC, median (IQR)	12 (7 to 16)	13 (8 to 17)	13 (9 to 18)	<0.0001
DOR, n/N (%)	223/1001 (22.28)	513/2711 (18.92)	188/1221 (15.40)	0.0002
TSH, mIU/L, median (IQR)	2.27 (1.67 to 2.95)	2.23 (1.64 to 2.96)	2.13 (1.52 to 2.81)	0.001
<2.5 mIU/L, n (%)	597 (59.64)	1633 (60.24)	795 (65.11)	0.007
≥ 2.5 mIU/L, n (%)	404 (40.36)	1078 (39.76)	426 (34.89)	
fT3, pmol/L, median (IQR)	4.59 (4.23 to 4.90)	4.79 (4.46 to 5.18)	4.96 (4.59 to 5.37)	<0.0001
fT4, pmol/L, median (IQR)	14.47 (13.75 to 14.97)	16.96 (16.17 to 17.79)	19.71 (19.13 to 20.62)	<0.0001
TAI, n (%)				0.4513
Positive	157 (15.68)	381 (14.05)	175 (14.33)	
Negative	844 (84.32)	2330 (85.95)	1046 (85.67)	
Infertility type, n (%)				<0.0001
Primary	366 (36.56)	1156 (42.64)	555 (45.45)	
Secondary	635 (63.44)	1555 (57.36)	666 (54.55)	
Infertility diagnosis, n (%)				0.0233
Tubal factor	564 (56.34)	1518 (55.99)	703 (57.58)	
Diminished ovarian reserve	203 (20.28)	470 (17.34)	186 (15.23)	
Male factor	72 (7.19)	249 (9.18)	122 (9.99)	
Unexplained	33 (3.30)	112 (4.13)	60 (4.91)	
Mixed factors	129 (12.89)	362 (13.35)	150 (12.29)	
Ovarian stimulation protocol, n (%)				0.0023
Long protocol	404 (40.97)	1206 (45.00)	579 (47.93)	
Antagonist protocol	369 (37.42)	1016 (37.91)	443 (36.67)	
Mild stimulation protocol	129 (13.08)	259 (9.66)	103 (8.53)	
Other protocols	84 (8.52)	199 (7.43)	83 (6.87)	
No. of aspirated oocytes, meidian (IQR)	9 (4 to 14)	10 (5 to 15)	11 (6 to 16)	<0.0001

AFC, antral follicle count; AMH, anti-Müllerian hormone; BMI, body mass index; fT3, free triiodothyronine antibody; fT4, free thyroxine; IQR, interquartile range; TAI, thyroid autoimmunity; DOR, diminished ovarian reserve

### fT4 concentrations and ovarian reserve markers in the study population

**Table 2:** fT4 concentrations and ovarian reserve in the included participants

Parameters	fT4 tertiles (pmol/L)			*P*-Value
	12-15.33 (*N* = 1001)	15.34–18.67 (*N* = 2711)	18.68-22 (*N* = 1221)	
AMH, ng/ml				
Adjusted mean [95%CI]	3.32 (3.16 to 3.50)	3.51 (3.40 to 3.62)	3.64 (3.50 to 3.80)	0.022
Mean difference [95%CI]	0.32 (0.04 to 0.59)	0.14 (-0.04 to 0.31)	Ref.	
*P*-Value	0.016	0.141	Ref.	
Risk of DOR				
n/N (%)	223/1001 (22.28)	513/2711 (18.92)	188/1221 (15.40)	0.0002
Adjusted OR [95%CI]	1.61 (1.01 to 2.58)	1.47 (1.00 to 2.16)	Ref.	0.085
*P*-Value	0.046	0.049	Ref.	
AFC				
Adjusted mean [95%CI]	12.3 (12.1 to 12.6)	12.6 (12.5 to 12.8)	12.7 (12.5 to 12.9)	0.039
Mean difference [95%CI]	0.4 (0.0 to 0.7)	0.1 (-0.2 to 0.3)	Ref.	
*P*-Value	0.046	0.521	Ref.	
NO. of aspirated oocytes				
Adjusted mean [95%CI]	9.7 (9.5 to 9.9)	9.9 (9.8 to 10.1)	9.9 (9.8 to 10.1)	0.140
Mean difference [95%CI]	0.2 (-0.1 to 0.5)	0.0 (-0.2 to 0.2)	Ref.	
*P*-value	0.173	0.940	Ref.	

AMH, anti-Müllerian hormone; DOR, diminished ovarian reserve; OR, odds ratio; AFC, antral follicle count

**Fig. 2:**
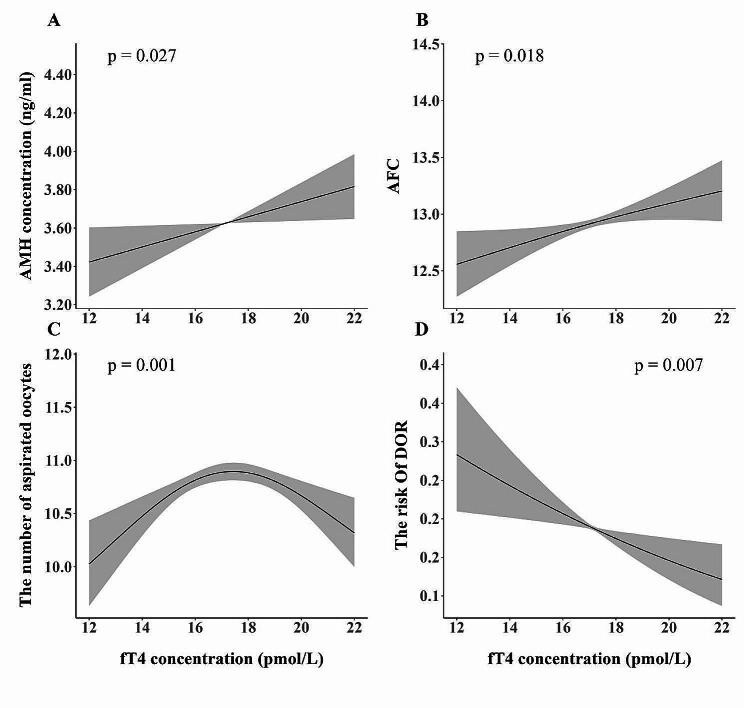
Associations between fT4 concentrations and ovarian reserve markers Association were analysed using generalised additive mixed models with smoothing splines. Solid lines indicate response curves, and dotted lines indicate 95% confidence intervals. **A**, AMH concentration; **B**, antral follicle count; **C**, the number of aspirated oocytes; **D**, the risk of diminished ovarian reserve

After adjusting for potential confounders, we found that the risk of DOR was higher in women in the lower (adjusted odds ratio [OR]: 1.61 [95% CI: 1.01 to 2.58]) and middle [1.47 (CI: 1.00 to 2.16)] tertiles than in those in the upper tertile, although the difference was not significantly different (*p* = 0.085). The AMH concentrations (adjusted mean: 3.32 [95% CI: 3.16 to 3.50] vs. 3.51 [3.40 to 3.62] vs. 3.64 [3.50 to 3.80] ng/mL, *p* = 0.022) and AFCs (adjusted mean: 12.3 [95% CI: 12.1 to 12.6] vs. 12.6 [12.5 to 12.8] vs. 12.7 [12.5 to 12.9], *p* = 0.039) were significantly different between the low-normal, middle-normal, and high-normal fT4 concentration groups. A lower adjusted mean value of the number of aspirated oocytes was observed in the lower fT4 tertile than in the middle and upper tertiles (adjusted mean: 9.7 [95% CI: 9.5 to 9.9] vs. 9.9 [9.8 to 10.1] vs. 9.9 [9.8 to 10.1]), although the difference was not statistically significant (*p* = 0.140) (Table 2). Notably, analysis using GAM with smooth curve fitting revealed that low-normal fT4 concentrations were associated with a decreased AMH concentration (*p* = 0.027), an increased risk of DOR (*p* = 0.007), a decreased AFC (*p* = 0.018), and a decreased number of aspirated oocytes (*p* = 0.001) (Fig. 2).

### fT4 concentrations and ovarian reserve markers in women aged < 35 and ≥ 35 years

To investigate whether a woman’s age would affect the associations of different fT4 concentration tertiles with ovarian reserve markers and the risk of DOR, we performed a subgroup analysis by age (< 35 and ≥ 35 years). Among women aged < 35 years, AMH concentrations were significantly different between the three groups (adjusted mean: 3.94 [95% CI: 3.70 to 4.20] vs. 4.25 [4.11 to 4.39] vs. 4.38 [4.18 to 4.58], *p* = 0.028). The risk of DOR was higher in the low-normal (adjusted odds ratio [OR]: 2.00 [95% CI: 0.96 to 4.18]) and middle-normal (adjusted OR: 1.57 [95% CI: 0.88 to 2.81]) fT4 concentration groups than in the high-normal group, although the difference was not significant (*p* = 0.158). However, there were no significant differences in the AFC or the number of aspirated oocytes among the three groups (*p* = 0.454 and *p* = 0.066, respectively). Among women ≥ 35 years, the AMH concentration was not significantly different among the three groups (*p* = 0.534). However, the risk of DOR was higher in the lower (adjusted OR: 1.51 [95% CI: 0.81 to 2.84]) and middle (1.46 [0.85 to 2.52]) tertiles than in the upper tertile, although the difference was not significant (*p* = 0.335). The lower fT4 concentration tertile had a lower AFC (mean difference = 0.7 [95% CI: 0.2 to 1.3], *p* = 0.005) than the higher fT4 concentration tertile. Moreover, the number of aspirated oocytes was significantly different between the three tertiles (adjusted mean: 6.5 [95% CI: 6.2 to 6.7] vs. 6.3 [6.2 to 6.5] vs. 6.7 [6.5 to 7.0], *p* = 0.024), as shown in Table 3.

**Table 3:** fT4 concentrations and ovarian reserve in women stratified by age

Parameters	fT4 tertiles (pmol/L)			*P*-Value
	12-15.33 (*N* = 1001)	15.34–18.67 (*N* = 2711)	18.68-22 (*N* = 1221)	
**Age<35** (***N*** = **3282)**	*N* = 598	*N* = 1797	*N* = 887	
AMH, ng/ml				
Adjusted mean [95%CI]	3.94 (3.70 to 4.20)	4.25 (4.11 to 4.39)	4.38 (4.18 to 4.58)	0.028
Mean difference [95%CI]	0.44 (0.05 to 0.83)	0.13 (-0.11 to 0.38)	Ref.	
*P*-Value	0.021	0.279	Ref.	
Risk of DOR				
n/N (%)	69/598 (11.54)	197/1797 (10.96)	86/887 (9.70)	0.472
Adjusted OR [95%CI]	2.00 (0.96 to 4.18)	1.57 (0.88 to 2.81)	Ref.	0.158
*P*-value	0.066	0.130	Ref.	
AFC				
Adjusted mean [95%CI]	14.5 (14.2 to 14.8)	14.7 (14.5 to 14.9)	14.7 (14.4 to 14.9)	0.454
Mean difference [95%CI]	0.2 (-0.3 to 0.7)	0.0 (-0.3 to 0.3)	Ref.	
*P*-value	0.648	0.919	Ref.	
NO. of aspirated oocytes				
Adjusted mean [95%CI]	11.7 (11.4 to 12.0)	12.1 (11.9 to 12.2)	11.9 (11.7 to 12.1)	0.066
Mean difference [95%CI]	0.2 (-0.2 to 0.6)	-0.2 (-0.5 to 0.1)	Ref.	
*P*-value	0.437	0.437	Ref.	
**Age ≥ 35** (***N*** = **1651)**	*N* = 403	*N* = 914	*N* = 334	
AMH, ng/ml				
Adjusted mean [95%CI]	2.28 (2.09 to 2.50)	2.20 (2.06 to 2.34)	2.32 (2.11 to 2.55)	0.534
Mean difference [95%CI]	0.04 (-0.26 to 0.33)	0.12 (-0.17 to 0.42)	Ref.	
*P*-value	0.946	0.946	Ref.	
Risk of DOR				
n/N (%)	154/403 (38.21)	316/914 (34.57)	102/334 (30.54)	0.093
Adjusted OR [95%CI]	1.51 (0.81 to 2.84)	1.46 (0.85 to 2.52)	Ref.	0.335
*P*-value	0.197	0.171	Ref.	
AFC				
Adjusted mean [95%CI]	8.8 (8.5 to 9.1)	9.1 (8.9 to 9.3)	9.5 (9.2 to 9.8)	0.005
Mean difference [95%CI]	0.7 (0.2 to 1.3)	0.4 (0.0 to 0.8)	Ref.	
*P*-Value	0.003	0.077	Ref.	
NO. of aspirated oocytes				
Adjusted mean [95%CI]	6.5 (6.2 to 6.7)	6.3 (6.2 to 6.5)	6.7 (6.5 to 7.0)	0.024
Mean difference [95%CI]	0.3 (-0.1 to 0.7)	0.4 (0.0 to 0.8)	Ref.	
*P*-Value	0.284	0.022	Ref.	

AMH, anti-Müllerian hormone; DOR, diminished ovarian reserve; OR, odds ratio; AFC, antral follicle count

**Fig. 3:**
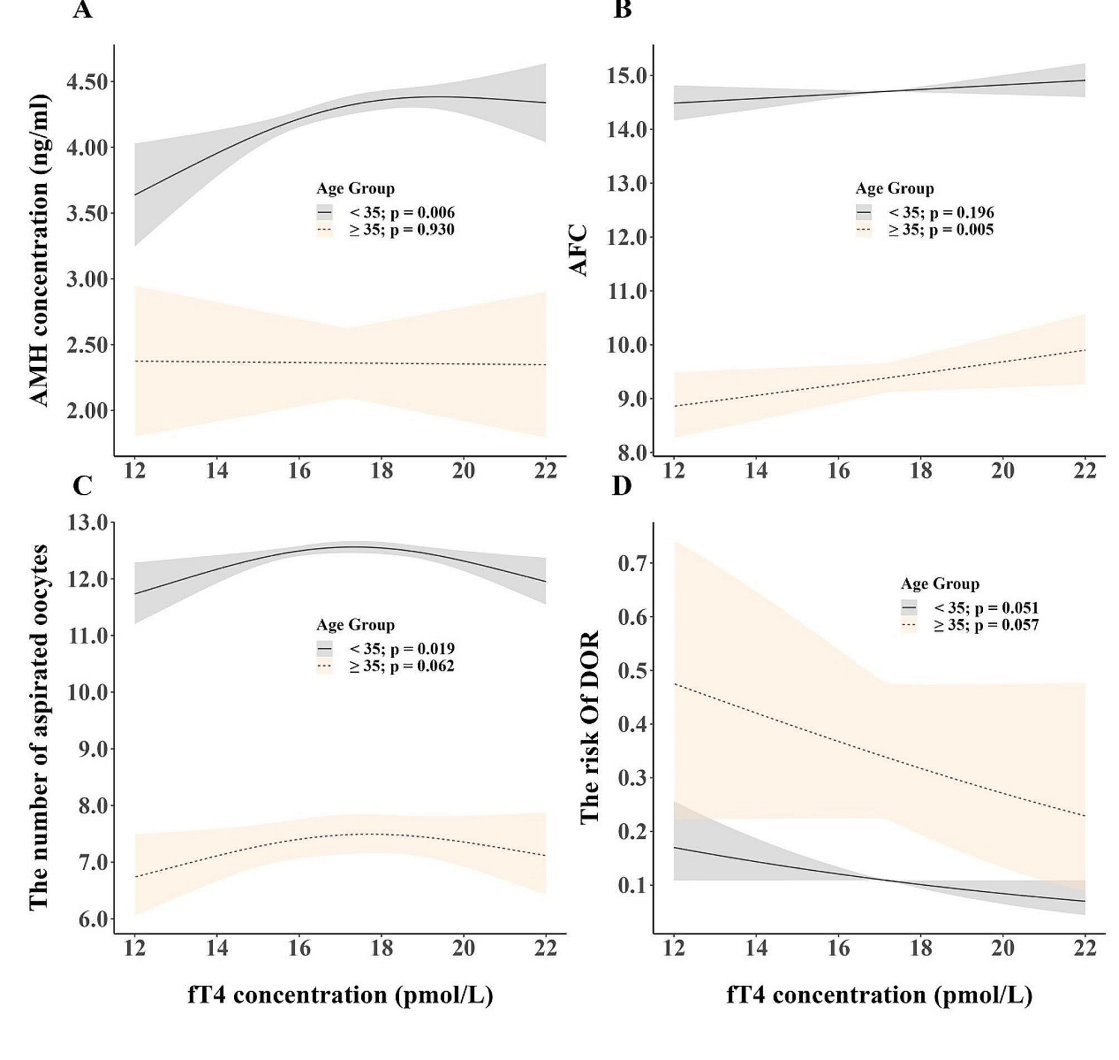
Associations between fT4 concentrations and ovarian reserve markers in women less than or over 35 years Associations were analysed using generalised additive mixed models with smoothing splines. Solid lines indicate response curves, and dotted lines indicate 95% confidence intervals. **A**, AMH concentration; **B**, antral follicle count; **C**, the number of aspirated oocytes; **D**, the risk of diminished ovarian reserve

Furthermore, the GAM using the fT4 concentration as a continuous variable showed that fT4 concentrations were positively and significantly associated with the AMH concentration (*p* = 0.006) and the number of aspirated oocytes (*p* = 0.019) in women aged < 35 years, but not in women aged ≥ 35 years (*p* = 0.930 and 0.062, respectively) (Fig. 3). Meanwhile, the fT4 concentration was not significantly associated with the risk of DOR in either age group (age < 35 years, *p* = 0.051; age ≥ 35 years, *p* = 0.057) (Fig. 3).In contrast, a significantly positive association was observed between the fT4 concentration and AFC in women aged ≥ 35 years (*p* = 0.005) but not in women aged < 35 years (*p* = 0.196).

## Discussion

This is the first study to investigate the association between a normal-range fT4 concentration and ovarian reserve in infertile women undergoing ART. The results demonstrated that women with a low-normal fT4 concentration had a lower AMH concentration and a higher risk of DOR. A further analysis indicated that the association between a lower fT4 concentration and lower AMH concentration was more obvious in women aged < 35 years than in those aged ≥ 35 years.

In contrast to the abundant evidence on associations between TSH concentration and reproductive function, the evidence on the impact of fT4 concentration on reproductive function is limited, especially on the impact of a normal-range fT4 concentration. However, after controlling for TSH concentrations within the normal range, we found that a lower fT4 concentration was associated with a lower AMH concentration, a lower AFC, and a higher risk of DOR. Existing studies have shown that thyroxine regulates ovarian development and function directly by binding to nuclear receptors or membrane proteins. First, thyroxine enters target cells by reversibly binding to different transporter proteins, such as thyroxin-binding globulin, or by diffusion or carrier-mediated transport involving membrane transporters. It then activates its nuclear receptors, such as the steroid receptor coactivator, and thereby stimulates or represses the transcription of target genes. Thyroxine also regulates ovarian function indirectly by activating a signal transduction cascade via the mitogen-activated protein kinase and extracellular signal-regulated kinase 1/2 pathways to regulate the transcription and phosphorylation of its nuclear receptors or through multiple interactions with other hormones and growth factors, such as estrogen, prolactin, and insulin-like growth factor [22]. Indeed, previous studies have reported that levothyroxine supplementation can improve ovarian function and reduce the risk of adverse clinical pregnancy outcomes in women with thyroid dysfunction [23–25], although the conclusion has not reached consensus.

As the regulatory mechanisms underlying the role of thyroid hormone in follicle development and ovarian reserve have not been fully elucidated, the following two aspects may contribute to the explanations of the results. First, thyroid hormones are critical for the development of follicles. Studies have reported that hypothyroidism inhibited the follicular development in rat models [26], possibly through the regulation of activity of nitric oxide synthase [27]. Propylthiouracil-induced hypothyroid rats presented relatively more atretic follicles and secondary, and less antral follicles relative to untreated rats [6]. In addition, thyroid hormones enhanced the effect of FSH in suppressing granulosa cell apoptosis and promoting granulosa cell proliferation via the PI3K/Akt pathway [28]. Since AMH is mainly synthesized and secreted by the granulosa cells from preantral and small antral follicles, the inhibited granulosa cell proliferation and increased apoptosis may inevitably impact the secretion of AMH. Low-normal FT4, although within the reference range, may reflect a subtle deficiency in thyroid function, and result in a decrease in ovarian reserve.

Ovarian reserve is known to decrease with advancing age, with 35 years being the commonly recognized cutoff age for reproductive aging [29]. Polyzos et al. reported that women older than 35 years are more likely to have a lower AMH concentration after radioactive iodine therapy [30]. However, in our study, a subgroup analysis based on age showed a positive association between fT4 and AMH concentrations in women aged < 35 years, whereas this association was not significant in women aged ≥ 35 years. These results suggest that we cannot ignore the adverse effect of a low-normal fT4 concentration on ovarian reserve. Notably, several studies have reported the influence of normal thyroid function on different diseases. For example, even normal-range variations in thyroid function affect serum cholesterol levels, blood pressure, and the risk of type 2 diabetes [11]. Normal-range variations in thyroid function have also been identified as risk factors for stroke and coronary artery disease [12]. Furthermore, we previously found that a low-normal paternal fT4 concentration was associated with a lower live birth rate in couples undergoing ART [13]. It is worth noting that these studies also remind us that the reference ranges for assessing thyroid function are based on the general population, and therefore, their application for infertile women remains questionable.

This study has several strengths. First, this study had a large sample size, and strict inclusion and exclusion criteria were applied. Second, all of the clinical data were collected from a single reproductive center, where the AMH test and thyroid function measurement were performed by three well-trained laboratory technicians and the transvaginal ultrasound detection procedures were performed by two well-trained reproductive endocrinologists, thus minimizing the inter-assessor variations. However, some limitations should also be noted when interpreting the results. First, given that the study population included infertile women who were undergoing ART treatment, the conclusions are not generalizable to the general population. Second, a causal relationship between the fT4 concentration and ovarian reserve in women could not be established due to the cross-sectional study design. Third, we did not consider potential genetic factors that may have affected ovarian reserve. Finally, the measurement of AFCs and the number of aspirated oocytes might have been influenced by different operators to some extent. Therefore, it is worth further investigating the relationship between a normal-range fT4 concentration and ovarian function through further studies.

In conclusion, this is the first study to investigate the association between a normal-range fT4 concentration and ovarian reserve in infertile women. The results showed that a low-normal fT4 concentration was associated with a lower ovarian reserve, particularly with a decreased AMH concentration, in women younger than 35 years. Future research involving the general population is warranted to further evaluate the associations between a normal-range fT4 concentration and ovarian reserve and to investigate the molecular mechanisms by which thyroid hormones regulate ovarian function.

## Data Availability

No datasets were generated or analysed during the current study.
